# Tracing reinforcement through asymmetrical partner preference in the European common vole *Microtus arvalis*

**DOI:** 10.1186/s12862-015-0455-5

**Published:** 2015-08-25

**Authors:** Mathias Beysard, Rebecca Krebs-Wheaton, Gerald Heckel

**Affiliations:** Computational and Molecular Population Genetics (CMPG), Institute of Ecology and Evolution, University of Bern, Baltzerstrasse 6, CH 3012 Bern, Switzerland; Swiss Institute of Bioinformatics, Genopode, CH 1015, Lausanne, Switzerland; Present Address: Max-Planck Institute for Evolutionary Biology, August-Thienemannstrasse 2, 24306 Ploen, Germany

## Abstract

**Background:**

The mechanistic basis of speciation and in particular the contribution of behaviour to the completion of the speciation process is often contentious. Contact zones between related taxa provide a situation where selection against hybridization might reinforce separation by behavioural mechanisms, which could ultimately fully isolate the taxa. One of the most abundant European mammals, the common vole *Microtus arvalis*, forms multiple natural hybrid zones where rapidly diverging evolutionary lineages meet in secondary contact. Very narrow zones of hybridization spanning only a few kilometres and sex-specific gene flow patterns indicate reduced fitness of natural hybrids and incipient speciation between some of the evolutionary lineages. In this study, we examined the contribution of behavioural mechanisms to the speciation process in these rodents by fine-mapping allopatric and parapatric populations in the hybrid zone between the Western and Central lineages and experimental testing of the partner preferences of wild, pure-bred and hybrid female common voles.

**Results:**

Genetic analysis based on microsatellite markers revealed the presence of multiple parapatric and largely non-admixed populations at distances of about 10 km at the edge of the area of natural hybridization between the Western and Central lineages. Wild females from Western parapatric populations and lab-born F1 hybrids preferred males from the Western lineage whereas wild females of Central parapatric origin showed no measurable preference. Furthermore, wild and lab-born females from allopatric populations of the Western or Central lineages showed no detectable preference for males from either lineage.

**Conclusions:**

The detected partner preferences are consistent with asymmetrical reinforcement of pre-mating reproductive isolation mechanisms in the European common vole and with earlier results suggesting that hybridization is more detrimental to the Western lineage. As a consequence, these differences in behaviour might contribute to a further geographical stabilization of this moving hybrid zone. Such behavioural processes could also provide a mechanistic perspective for frequently-detected asymmetrical introgression patterns in the largely allopatrically diversifying *Microtus* genus and other rapidly speciating rodents.

## Background

The mechanisms by which speciation progresses and is completed remain elusive for most organisms. Separation in allopatric ranges provides a plausible condition where selection and/or genetic drift may lead to differences in morphological, physiological or behavioural phenotypes which increase reproductive isolation between taxa and thus promote speciation. It has been argued, though controversial, that pre-zygotic isolation could be reinforced when two taxa have returned to parapatry and experienced hybridization [1–6]. If the outcome of cross-fertilization between incipient species results in unfit hybrids, natural selection could favour the reinforcement of pre-zygotic isolation to avoid costly maladaptive hybridization [2, 3, 7, 8]. The progressive reinforcement of pre-zygotic isolation mechanisms could ultimately achieve speciation, thus fully isolating the two taxa [2, 8]. This holds true if the cost of hybridization is symmetrical, with sufficient selection pressure acting on both taxa involved. In the presence of asymmetrical maladaptive hybridization ([9 and references therein, 10, 11]), the reinforcement process is expected to evolve in an analogous asymmetrical manner. Yukilevich [12] has recently shown in a meta-analysis of species pairs of *Drosophila* that the direction and the strength of hybrid dysfunction are indicative of the direction of reinforcement, suggesting that asymmetrical post-zygotic isolation could lead to a concordant increase of pre-zygotic isolation.

The rodent genus *Microtus* has probably experienced the fastest radiation in extant mammals in the last 1.5 million years [13]. Strong genetic differentiation within many nominal species suggests ongoing speciation processes with different levels of reproductive isolation or the presence of cryptic species [14–16]. In the common vole (*Microtus arvalis*), four main parapatric evolutionary lineages (Western, Central, Italian and Eastern) defined by mtDNA, Y-chromosomal and autosomal DNA are spread across Central Europe with additional lineages (Balkans, *M. obscurus*) in the east [17–23]. The origin of the divergence between these evolutionary lineages probably dates back to the Last Glacial Maximum [24]. The recolonization of Europe from their allopatric glacial refugia has led to multiple secondary contacts where hybrid zones have formed which differ in the age of divergence between the evolutionary lineages involved and the level of gene flow between them [18–20, 25]. Investigations of the sex-specific genetic structure of these hybrid zones have shed light on ongoing speciation processes between the *M. arvalis* lineages, with indirect evidence for partial reproductive isolation between the Central, Western and Italian lineages [20, 25]. The absence of Y-chromosome introgression between the Western and Central lineages relative to autosomal markers despite male-biased dispersal in the species [26–28] and the very narrow area of hybridization (a few kilometres) support a lack of fitness at least for some male hybrids in their natural environment [25]. We have suggested that hybridization should be more detrimental to the Western lineage, as this lineage lost ground to the Central lineage since the initial secondary contact but it remains unknown if potential asymmetrically-acting pre-mating mechanisms are involved in the dynamics of the hybrid zone [25].

The specific structure and dynamics of the hybrid zone between the Western and Central lineages of the common vole offer the opportunity to study the role and evolution of pre-mating mechanisms across a secondary contact. If hybridization has resulted in the establishment of specific asymmetrical partner preferences, these could have arisen relatively soon after admixture [5] and should be detectable in populations close to the centre of hybridization. By comparing the partner preference of females from parapatric populations in the hybrid zone to the preferences of females from allopatric regions, it is possible to assess if pre-mating isolation evolved potentially in reaction to maladaptive hybridization or if it existed before the secondary contact. We focus here on female rather than male partner preference because females are usually considered to be the sex with higher physiological costs of reproduction in mammals and should thus be choosier in mate choice [29, 30], and sex-specific gene flow patterns in the Western-Central hybrid zone of the common vole suggest selection in particular against males [19, 25].

In this study, we assess directly the preferences of female common voles from populations across the zone of asymmetrical hybridization for males from the Western or Central evolutionary lineages. Nuclear genetic markers were used to determine the extent of the area of hybridization and to localise parapatric, non-admixed vole populations. To detect potential reinforcement of partner preferences in the area of hybridization, we tested then experimentally the partner preferences of females from these parapatric populations for males from the two evolutionary lineages, and compared this to the preferences of females from allopatric populations outside of the area of recent and past hybridization. The partner preferences of the wild individuals were further compared to those of lab-born females derived from allopatric populations in the non-admixed ranges of the Western and Central lineages and those of first generation hybrids.

## Results

### Parapatric and allopatric populations in the hybrid zone area

**Fig. 1:**
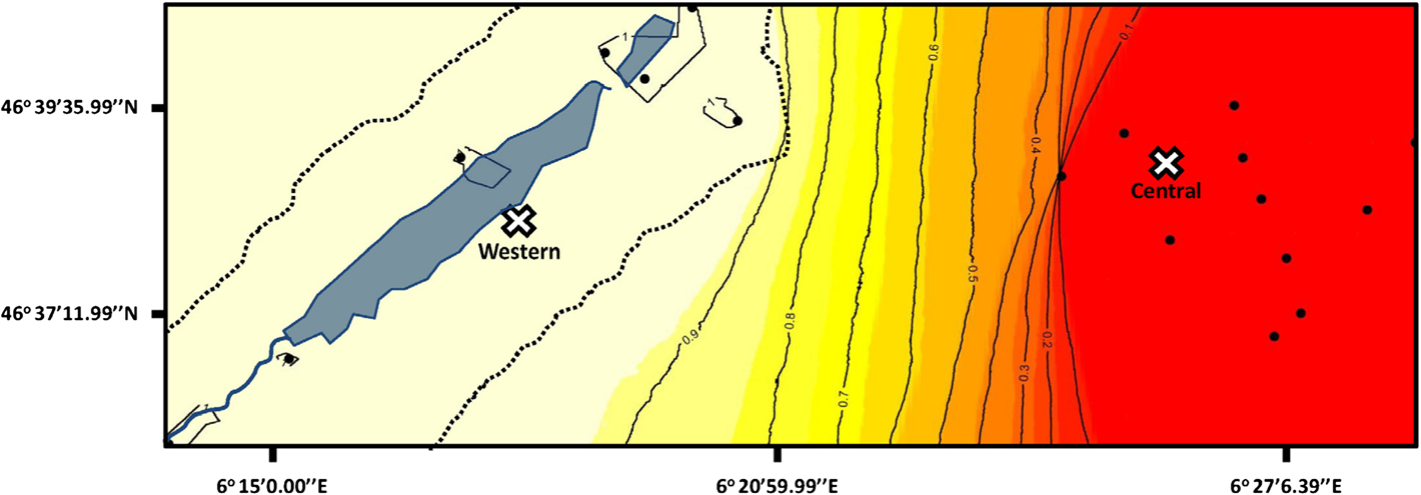
Genetic background and localisation of the parapatric *Microtus arvalis* populations (crosses) analysed in the hybrid zone between the evolutionary lineages Western and Central. The probability of membership for genetic clusters obtained with Geneland is given in light yellow (>0.9) for the Western and in red (<0.1) for the Central lineage. The distance between the two experimental populations is 8.4 km. Black dots represent other populations included in the admixture analyses but not in tests of partner preferences. The allopatric populations for partner preference tests were localised farther to the west and east of the area shown. The lakes and stream in the valley are represented in blue, while the crests of the valley are the dotted lines

Dedicated sampling provided a refined characterization of the zone of admixture between the Western and Central evolutionary lineages in the Swiss Jura mountain range. Targeted trapping around the centre of the hybrid zone yielded 60 individuals additional to the 371 described in Beysard & Heckel [25] for which genotypes were obtained at 14 highly variable microsatellite loci. Bayesian clustering analyses of all 431 individuals showed the presence of the two pure evolutionary lineages at localities of less than ten kilometres distance on either side of the crest of a valley (Figure 1). Based on the genetic landscape map and admixture proportions smaller than 0.1 at the localities, we then selected populations (distance: 8.4 km) on either side of the nuclear admixture area for the partner preference tests of females of parapatric origin. We obtained 17 females from the Western parapatric population and 14 females from the Central parapatric population for preference tests. These were complemented by individuals from allopatric populations (17 and 15 females, respectively) farther outside of the area of hybridization (distance: 64.2 km).

### Partner preference tests

**Fig. 2:**
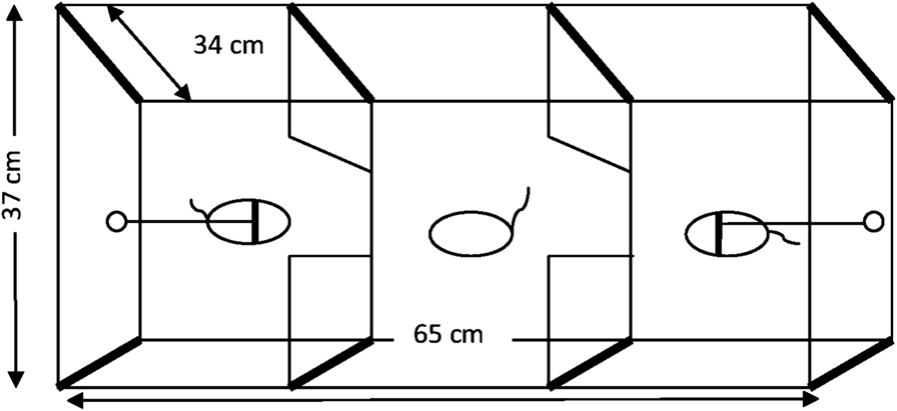
Experimental setup for the evaluation of female partner preference in common voles (view from above). A plastic box was divided into three chambers by opaque plastic rectangles with a gap in the middle which allows the female to visit the stimulus males. The central chamber is inaccessible to the leashed males, providing therefore a safe zone for the female. The setup was recorded by a video camera 1 m above the central chamber

Females spent their time in the preference test mostly with unfamiliar and unrelated males from the Western or Central lineages rather than alone in the central compartment of the apparatus (Fig. 2). All females visited both males in the respective test already during the initial discovery time. Also thereafter, females had always the opportunity to avoid social contact and remain in the central compartment without being seen by the males, but the proportion of time spent in the central compartment was low (13.8 % on average, sd = 0.22). The experimental situation appeared not to prevent normal social behaviour of males, since they showed typical signs of interest in the visiting females by sniffing, licking, following them around, huddling and sometimes mounting them. Some huddling periods and other social interactions were followed by repeated mating in the test apparatus.

**Fig. 3:**
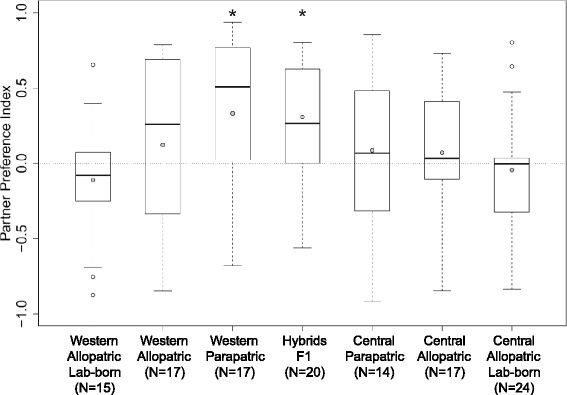
Partner preference index (PP_ind_) of female *M. arvalis* of allopatric, parapatric or hybrid origin for males from the Western or Central lineages. A PP_ind_ of 1 would indicate a complete preference for Western males, −1 a complete preference for Central males and 0 the absence of a preference. Each female category is represented by a boxplot, the bold line being the median and the grey circle being the mean. A star above a boxplot marks a significant deviation from zero

Our analyses showed that partner preference of female common voles varied according to their origin across the zone of admixture in nature (Fig. 3). Time spent with the males was quantified with the partner preference index (PP_ind_) where 1 would indicate a complete preference for a Western male, −1 a complete preference for a Central male and 0 would indicate an absence of preference (see Material and Methods). The Western females of parapatric origin showed a strong preference for Western males (mean PP_ind_ = 0.33; *p* =0.02, *N* = 17). Western females from allopatric populations chose a male regardless of their lineage of origin (wild: mean PP_ind_ = 0.12; *p* = 0.35, *N* = 17, lab-born: mean PP_ind_ = − 0.11; *p* = 0.39, *N* = 15). Partner preferences of the wild Western females from parapatric populations were significantly stronger than those from allopatric populations (Mann–Whitney test, *p* = 0.04). No significant preference for Western or Central males was detected in the Central females from wild parapatric (mean PP_ind_ = 0.06; *p* = 0.89, *N* =14) or allopatric populations (mean PP_ind_ = 0.07; *p* = 0.63, *N* = 15) or in the lab-born Central females of allopatric origin (mean PP_ind_ = − 0.04; p = 0.59, *N* = 24). However, a strong preference for Western males was also detected in the F1 hybrid females (mean PP_ind_ = 0.3; *p* =0.002, *N* = 20). Ten of these females were maternally of Central and ten of Western origin but only a single individual showed a preference (PP_ind_ = −0.56) for a Central male.

## Discussion

Our analyses show a stronger partner preference of female common voles from the Western edge of the secondary contact zone compared to females of allopatric origin. This is consistent with a signature of asymmetrical reinforcement of partner preference, suggesting that premating isolation mechanisms did not evolve in allopatry but after hybridization in secondary contact. The presence of a similar pattern among first generation hybrid females supports a potential role of the hybridization process in the evolution of partner preference in the common vole.

### Signature of reinforcement of pre-mating mechanisms

Previous investigations of the secondary contact zone between the evolutionary lineages Western and Central in *M. arvalis* revealed a very narrow area of current hybridization, which coupled with an absence of Y-chromosome introgression compared to other parts of the genome, suggests a lack of fitness for some hybrid males [25]. The extended data set analysed here confirmed the localisation of the narrow area of contact. The current position of the hybrid zone is the result of the replacement of the Western lineage by the Central lineage, likely due to an asymmetric deficit of fitness afflicting the Western lineage [25]. If post-zygotic isolation affects one of the taxa more, this taxon is expected to exhibit stronger reinforcement [12]. Thus, the detection of a stronger preference for Western males in the Western parapatric population is consistent with an evolutionary response to asymmetrical maladaptive hybridization and may represent an asymmetrical signature of reinforcement of pre-zygotic isolation.

It has been argued that reinforcement requires some gene flow, but that an excess of it could erode its effects ([5, 31] and references therein). In the present situation, it is possible that the asymmetrical dynamics of gene flow detected with autosomal, Y-chromosomal and mtDNA markers at the contact zone [25] have provided the conditions for reinforcement in the Western population only. After initial secondary contact and local replacement of the Western lineage, the advance of the Central lineage was apparently stopped in the Vallée de Joux, resulting in a narrow area of admixture on its inner slope [25]. Thus the tested Central voles from the edge of the admixture area on the outer slopes of the valley might not have enough contact at present with Western individuals to induce or maintain reinforcement. Hybridization during the initial contact of the *M. arvalis* lineages in the area might have potentially led also to a reinforcement of the partner preference of Central females. However, incoming migrants from the non-reinforced Central populations would lead to a dilution of the effects of reinforcement until disappearance [5].

Comparable studies on other rodents are very rare despite their enormous number of species and rate of evolutionary diversification. A case of apparently similar asymmetrical reinforcement was detected in the European house mouse hybrid zone, where maladaptive hybridization between subspecies is present but varies strongly according to geography [32–34]. Evidence for reinforcement in the house mouse system stems also from the border of the hybrid zone, where it was shown that urinary signals permit *Mus musculus musculus* individuals to avoid mice from a *M. m. domesticus* or hybrid origin [6, 35–37]. Ganem and colleagues [38] suggested that the dominant behaviour of *domesticus* males over *musculus* males may have resulted in asymmetrical effective migration favouring asymmetrical reinforcement in the hybrid zone, and differences in explorative behaviour have been described [39]. The data presented here do not allow us to address a potential role of dominant behaviour of Central or Western males in the detected pattern of asymmetrical reinforcement, but this factor could be tested in the future.

### Conditions for establishing reinforcement

The outcome of hybridization often shows intermediate traits compared to the parents, but hybridization has also the potential to be a creative force leading via transgressive segregation to evolutionary novelties like different behaviours or other new phenotypes in hybrids ([39], recently reviewed in 40). In the context of mate preferences, hybrids may then favour or ignore different traits compared to the parents, which might explain the strong partner preference of the first-generation hybrids for Western *M. arvalis* males (Fig. 3). In the house mouse system, Christophe and Baudoin [41] found overall a significant preference of lab-born F1 hybrid females for *musculus* males, and wild caught hybrids may also show a preference for *musculus* traits [37]. On the mechanistic side, our results could be explained parsimoniously by epistasis involving at least two loci. An allele of Central origin would activate a preference for Western males by interacting with an allele of Western origin at a different locus. This would also permit the fixation of this Central allele at the Western edge of the contact zone by repetitive backcrosses, consistent with the observed pattern. Recent studies in the house mouse system have shown that epistatic genes could indeed be involved in reproductive isolation mechanisms at the post-and pre-zygotic level [42].

### The impact of partner preference on the dynamics of the hybrid zone

The movement of the Western-Central hybrid zone in *M. arvalis* was hypothesized to have been driven by particular partner preference in addition to asymmetrical post-zygotic isolation [25]. Two different kinds of partner preferences could lead to a replacement of one taxon by another: an asymmetric conspecific mate preference or a particular preference for non-conspecifics (reviewed in [43]). For example, *Orchelimum nigripes* katydid females prefer to mate with conspecific males which results in the replacement of the non-discriminating *O. pulchellum* [44]. On the contrary, the movement of a water strider hybrid zone is likely driven by a strong preference of female *Limnoporus notabilis* for male *L. dissortis* [45]. In the common vole, the preference of female F1 hybrids for Western males is at odds with the direction of movement of the contact zone detected in Beysard & Heckel [25]. Overall, the absence of a detectable preference for any lineage in the allopatric voles does not support a key role for partner preferences in the replacement of the Western by the Central lineage since this would probably require a particular partner preference also in pure populations. Nevertheless, the existence of a preference for Western males in the Western population at the edge of the contact zone might contribute to the stabilization of the hybrid zone, preventing (or slowing down) further advancement of the Central lineage.

Given the polygynous mating system of the common vole with frequent multiple paternity [46, 47], the influence of mate choice of female *M. arvalis* on realized reproduction should be considered. Multiple paternity might result from coercive mating or from a female’s choice of one (or several) other partners with favourable traits [48, 49]. However, successive mating with different males may lead to sperm competition, another pre-zygotic mechanism potentially involved in reproductive isolation and the dynamics of secondary contact ([50 and references therein, 51]). Asymmetries in fertilization success of sperm from males from the parental lineages or an advantage of sperm from males of pure parental lineages over hybrids (see [51]) may at least contribute to the structure and dynamics of gene flow in the hybrid zones of *M. arvalis* and form testable hypotheses for future analyses.

## Conclusions

The detected partner preferences in female common voles are consistent with asymmetrical reinforcement of pre-mating reproductive isolation mechanisms. As a consequence, these differences in behaviour might contribute to a reduction in gene flow between the evolutionary lineages and a further geographical stabilization of this particular moving hybrid zone (see also [25]). However, if specific partner preferences may evolve rapidly after secondary contact and hybridization between allopatric rodent lineages, similar processes could also provide a mechanistic perspective for the asymmetrical introgression patterns that have been detected in multiple other *Microtus* taxa (e.g. [14–16] and references therein). The contribution of such processes to the extremely rapid rates of speciation in *Microtus* and many other groups of rodents [52] thus deserves further exploration.

## Materials and methods

### Admixture in the common vole hybrid zone

In order to precisely test voles from populations at the edge of the area of hybridization where pre-mating isolation would be most relevant, we extended the sampling around the centre of recent hybridization described in Beysard & Heckel [25] by genotyping 60 newly trapped individuals (added to an initial data set of 371 voles) with 14 microsatellite markers [53]. We then ran Geneland 2.0.12 [54] to obtain a detailed description of the distribution of the lineages in the geographic centre of admixture. Analogous to Beysard & Heckel [25], we assumed two genetic clusters and performed 10 runs of 1 000 000 iterations with 50 000 burn-in. After checking for consistency between the 10 runs, we displayed the run with the best likelihood on a map of probability of membership for each lineage (Fig. 1).

### Localization of parapatric and allopatric populations

We then selected sites at the edge of the nuclear admixture area to trap females for the partner preference tests. These sites were the first suitable habitats outside the area of detectable nuclear admixture which showed signs for the presence of many voles (numerous burrows). We refer to these two sites as Western and Central parapatric populations. The Western parapatric population (17 females) was located on the inner slope of the Vallée de Joux (46°38'12"North, 6°17'53"East) whereas the Central parapatric population (14 females) was on the outer slope (46°39'13"North, 6°24'47"East). For a comparison with allopatric females, we selected populations outside of the area of recent and past hybridization (allopatric Western population (17 females) 46°41'11"North, 6°8'46"East; allopatric Central population (15 females) 46°35'34"North, 6°34'36"East; see [25]). Voles were trapped with Longworth small mammal traps (Penlon).

### Experimental animals

Animal experimentation for this study followed the guidelines of the Association for the Study of Animal Behaviour and trapping occurred under permits BE-08/10 and BE-90/10 issued by the cantonal veterinary offices after approval by the Bernese cantonal commission on animal experimentation. We were committed to reduce stress for the animals as much as possible. Voles were kept before partner preference tests in 17 x 28 x 13 cm polycarbonate cages (Indulab, Gams Switzerland) with a thick layer of wood chips and paper tubes as structural enrichment. They were supplied with rodent pellets (Provimi Kliba, Kaiseraugst Switzerland), carrots and water *ad libidum*. The room was maintained on a 14:10 light:dark schedule at an ambient temperature of 21 °C. Wild-caught voles were acclimatized for at least two weeks before partner preference tests. During the acclimatization time, potential ecto- and endo-parasites were eliminated with Ivomec (Merial, Derendingen Switzerland) in order to avoid infection during the experiments.

Partner preferences of wild females were compared to lab-born females which were derived from allopatric populations in the non-admixed ranges of the Western and Central lineages. The Western voles stemmed from several populations of the Département du Jura and Département de Saone-et-Loire (France) and the Central voles from various populations in the cantons Bern and Schaffhausen (Switzerland). We tested unrelated (i.e. stemming from crosses with different parents and no known kin-relationship) virgin lab-born females after the age of 40 days. Since female common voles are precocious breeders and are capable of reproducing before they are weaned (14–18 days old), we were certain that the females were of reproductive age [55]. The lab born females were either pure Central (first or second generation, 24 individuals), pure Western (first or second generation, 15 individuals), or F1 hybrids between pure Central and Western lineages (20 individuals). Among these F1 hybrids, ten females were offspring from crosses between Central males and Western females and the others from the reverse combination.

The males used as stimuli for the partner preference tests were first or second generation lab-born from the same pure allopatric Central and Western stock but unfamiliar and unrelated to the experimental females and to each other (i.e. no close kin relation). Males were kept with their siblings until 30 days of age and were then housed individually to avoid the establishment of dominance behaviour, which might influence partner preference tests [56].

### Partner preference testing setup

We used an experimental setup for the partner preference tests that is widely-used for assaying social and sexual behaviour in rodents (see [57] and references therein). The setup consisted of a plastic box (65 cm x 37 cm x 34 cm) divided into three chambers by opaque plastic rectangles, with a gap in the middle which allows the female to travel between the chambers (Fig. 2). Stimulus males were prevented from entering the central compartment by a collar around the neck which was attached to their respective compartments at the short sides of the box with steel fishing wire (Flexonit 0.45 mm diameter, Cebra Plochingen, Germany). The central compartment was accessible only to the females and provided them a non-choice/safe zone. The voles were provided with water in sipper tubes and food pellets in all three compartments. The floor was covered by a layer of wood chips. Boxes were cleaned after each experiment and washed with ethanol (70 %).

Stimulus males were placed in the apparatus three hours prior to the introduction of the experimental female in order to give them time to adjust to the situation and explore their compartment [57, 58]. Females were introduced to the central compartment, and behaviour was recorded for 4.5 h using a Logitech C510 camera positioned 1 m above the centre of the experimental setup. The first 30 min were considered as discovery time to allow the females to explore the test apparatus and not scored. Videos were recorded in .mov format and behaviour was manually scored using JWATCHER v1.0 [59].

Preference of a female for a male was quantified as the proportion of time spent huddling. Huddling was defined as close, physical, predominantly immobile or affiliative contact [57]. This measure has been shown to be the most sensitive indicator of a partner preference in *Microtus* species [57, 60, 61]. The partner preference index (PP_ind_) was calculated as follows:

- PP_ind_ = (Time huddling with Western -Time huddling with Central)/Total test time

A PP_ind_ of 1 would indicate a complete preference for a Western male, −1 a complete preference for a Central male and 0 would indicate an absence of preference. Note that any time spent by the female in the central compartment of the test apparatus decreases the maximum value of the PP_ind_ accordingly but this reached on average only 13.8 % of the test time. The deviation of the median from zero was tested with a Wilcoxon test for each female category in R.

## Availability of supporting data

The original dataset supporting the results of this article is available on Dryad http://dx.doi.org/10.5061/dryad.8n2f2. Further information is available from the authors upon request.

## Abbreviations

F1: First generation
mtDNA: Mitochondrial DNA
PP_ind_: Partner preference index
sd: Standard deviation
